# Perceptions and experiences of Latinx parents with language barriers in a pediatric emergency department: a qualitative study

**DOI:** 10.1186/s12913-022-08839-w

**Published:** 2022-12-01

**Authors:** Ronine L. Zamor, Lisa M. Vaughn, Erin McCann, Luisanna Sanchez, Erica M. Page, E. Melinda Mahabee-Gittens

**Affiliations:** 1 Division of Emergency Medicine, Cincinnati Children’s Hospital Medical Center, 3333 Burnet Avenue, Cincinnati, OH 45229 USA; 2 Department of Pediatrics, University of Cincinnati College of Medicine, 3230 Eden Avenue, Cincinnati, OH 45267 USA; 3 Present Address: Division of Emergency Medicine, Children’s Healthcare of Atlanta, Emory University, 1547 Clifton Road, NE 2nd Floor, Atlanta, GA 30322 USA; 4 Department of Pediatrics, Cincinnati Children’s Hospital Medical Center, 3333 Burnet Avenue, Cincinnati, OH 45229 USA

**Keywords:** Latinx, Pediatric emergency department, Language barriers, Health disparities

## Abstract

**Background:**

Prior research has shown disparities exist among Latinx children who require treatment for respiratory illnesses within the pediatric emergency department (PED). Limited data exist regarding Latinx families’ experiences on the care they received at PEDs within non-traditional destination areas (NDA). Their experiences can identify areas of improvement to potentially reduce healthcare disparities among pediatric patients within this population. The purpose of this qualitative study was to explore the lived experiences of Latinx families with low English proficiency in the PED with a NDA. The broader purpose was to identify areas of improvement for reducing health care disparities among Latinx families.

**Methods:**

We used qualitative methods to analyze semi-structured interviews among Latinx families who presented to the PED with their 0–2 year-old child for a respiratory illness from May 2019 through January 2020. All participants had low English proficiency and requested a Spanish interpreter during registration. All interviews were transcribed and reviewed using thematic analysis based on a phenomenology framework.

**Results:**

Interviews were conducted with 16 Latinx parents. Thematic analysis revealed four major themes: (1) Uncertainty - Families expressed uncertainty regarding how to care for a child with distressing symptoms, (2) Communication – Families favored in-person interpreters which enhanced communication and allowed families to feel more informed, (3) System Burden – Families reported that the unfamiliarity with the US health system and lack of resources are additional burdens, and (4) Emotional Support – The emergency department visits garnered confidence and reassurance for families.

**Conclusions:**

Our study identified four major themes among Latinx families within a PED of a NDA. Potential areas of interventions should focus on supporting access to an interpreter, improving information delivery, and enhancing education on community resources for families with low English proficiency.

## Background

In 2016, the Census Bureau estimated that the Hispanic population of the United States (U.S.) was 56.6 million, which constitutes 17% of the nation’s total population. This proportion is expected to increase to 28% by 2060 [1]. About one-third of this population are children, which is the greatest proportion of children compared to proportions among families of Black, Asian or White origin [2, 3]. In the past, immigration of Latinx families to the U.S. has largely been concentrated in states with large urban areas, such as Texas, Florida, and New York [4]. However, recent growth among the Latinx population has been observed in areas of the U.S. that were not common destination areas in the past, such as areas within the Midwest, which are defined as nontraditional destination areas (NDAs) [5]. NDAs, such as Ohio and Nevada, have experienced at least between 100 and 200% growth in the past 2 decades compared to traditional areas, which experienced at least a 20% drop [6, 7]. The rapid growth of the Latinx population has significant health implications because NDAs do not have the infrastructure and resources to support the changing immigration patterns [6, 8]. For instance, a prior study conducted in the Midwest showed that linguistically and culturally appropriate services to meet the need of the growing Latinx community are lacking [5]. However, literature describing Latinx healthcare experiences within NDAs remains limited.

Prior literature has shown significant healthcare disparities among Latinx children whose families face language barriers within the emergency department (ED) [9–12]. For instance, there are differences in diagnostic testing and hospital charges in the pediatric emergency department (PED) among families with language barriers [9, 13, 14]. Some studies suggest that language barriers may be associated with poor health outcomes such as increased obesity and complications of appendicitis [10, 15, 16]. Compared to Latinx patients in traditional destination areas, Latinx patients in NDAs have different healthcare barriers, experiences, and needs because healthcare systems in NDAs are not properly equipped to work with populations that have limited English proficiency [5, 6, 17–19]. Latinx patients in NDAs may face more challenges such as social isolation, discrimination, and lack of social support [5, 6, 18, 20]. Given the rapid growth of Latinx population and the need for promoting greater equity in pediatric emergency care [21], exploring the experiences and perceptions of Latinx families surrounding their management in the PED can help target areas of improvement for reducing disparities within a NDA. Given the known disparities among pediatric patients with respiratory complaints [9], the purpose of this study is to explore the lived experiences of non-English speaking Latinx families with children with respiratory illnesses within a PED of a non-traditional destination area. The broader aim of this study is to identify areas of improvement for reducing health care disparities for Latinx families.

## Methods

### Study setting

The study was conducted at Cincinnati Children’s Hospital Medical Center (CCHMC), one of the largest academic children’s hospitals in the United States. The hospital treats children with rare and complex conditions from over 60 different countries [22]. There are two different PEDs which in combination have approximately 92,000 visits per year. Upon arrival to the PED, families are asked their preferred language and whether they need an interpreter for the remainder of the visit. The preferred language is documented within the patient’s chart. An interpreter can be used at any time or for the entire visit depending on availability, which includes access to an interpreter in-person, via video call, or via telephone.

### Recruitment

We conducted purposive sampling of patients by screening electronic health records (EHR) for potential participants 7 day a week from May 2019 through January 2020. Parents or legal guardians were considered eligible for the study if they: 1) presented to the PED with their 0–2-year-old child for a viral upper or lower respiratory illness, 2) affirmed that their preferred language was Spanish at triage, and 3) requested a Spanish interpreter during registration. At the time of this study, only one pediatric emergency medicine attending was considered a certified language interpreter in Spanish. All other physicians were required to use an interpreter if the patient and/or family did not speak English.

Potentially eligible families were approached by study staff while patients were in the PED. The study staff and a Spanish interpreter explained the study, review informed consent, and scheduled a telephone interview once informed verbal consent was obtained.

Study staff also conducted weekly screening of the EHRs to identify potentially missed eligible families who had already been discharged. If the family met the inclusion criteria, study staff called the family to inform them of the study, solicit participation, and obtain informed verbal consent. The patient’s age, medical history, main complaint, and type of interpreter involved with the family was reviewed and confirmed prior to proceeding with the interview. All participants were given a $20 gift card in exchange for their participation in the study.

### Data collection

Three authors conducted 16 semi-structured, one-on-one qualitative interviews from May 2019 through January 2020. Two (L.S. and E.M.) of the three interviewers were fluent in Spanish and did not use an interpreter for the interviews. One interviewer (L.S.) was of Hispanic descent. The other two interviews (E.M and R.Z) were not of Hispanic descent, however both have worked primarily with Latinx patients during their medical training. All interviewers received qualitative interview training from L.V. who has doctoral level training and research experience in qualitative research methods within the Latinx community. The interviewers used an interview guide (Table 1) that allowed them the flexibility to deviate from the guide and ask probing questions based on participants’ responses.

**Table 1:** Interview guide

1. Where are you from? How long have you lived in the United States?	8. Was there anything done that day that you think helped your child feel better?
2. What resources do you use in your community when your child is sick?	9. Describe your understanding of why things were or were not done.
3. If you remember, what brought you and your child to the emergency department?	10. How did your doctor communicate with you when deciding what should or should not be done?
4. What concerned you the most about your child’s symptoms when you visited the emergency department?	11. Describe how you communicated what concerned you regarding your child during your visit.
5. Do you remember if you took your child to his/her primary doctor before going the emergency department that day? Tell me about that.	12. What things did you not tell your doctor that you wish you had told them?
6. Tell me what the several days before you took your child to the emergency department were for you.	13. How did you feel having an interpreter there helped or did not help with your visit that day?
7. Describe to me how your visit went. Ex: Testing, treatments, expectations	14 How do you think communication about what was done during your visit could have been better?

All families who gave informed verbal consent while in the PED had their interview completed via phone within 30 days of the index PED visit. All interviews were audiotaped then translated and transcribed. Interviews were conducted until thematic saturation was reached, which occurred when the interviewers did not elicit any new information or themes [23].

### Data analysis

This study drew from the phenomenology framework developed by Max Van Manen and Martin Heidegger, who described a hermeneutic approach to phenomenology that stresses how human experiences and “realities” are influenced by the world in which they live. This framework provided a way to understand each family’s meaning that they constructed from their experiences in the PED [24–26]. The research team utilized a thematic analysis approach to systematically identify meaningful subthemes and themes that represented major topics discussed in the interviews [27, 28]. Thematic analysis involved an iterative process to further clarify the themes that could be presented in a cohesive narrative. First, the research team independently reviewed all interview transcripts and developed codes, which were key words that represented specific phrases, patterns, and common features found in the transcripts. A codebook was developed to help guide the data analysis. All members of the research team collectively reviewed and coded all transcripts. Three other researchers other than the interviewers were included in the analysis for investigator triangulation. The research team met regularly to review the transcripts and discuss emerging codes until code agreement and saturation were reached. Each code was collated into unifying themes along with subcategories within each theme. The software program *Dedoose* was used to highlight specific themes or common phrases that might have been missed when reviewing the transcripts as a group [29]. Code frequencies within *Dedoose* were also used to identify patterns in families’ responses [29].

## Results

### Characteristics of study participants

Interviews were conducted with 16 Latinx parents (15 mothers, 1 father) who were seen in the PED with a child presenting with respiratory symptoms. The length of interviews ranged from 11 minutes to 26 minutes (median, 20 minutes). Most pediatric patients were male (66%). Among the families who were comfortable sharing their country of origin and length of time in the US, the range of time in the US ranged from 18 months to 13 years. Table 2 shows the proportions of patient triage levels, chief complaints, disposition, and final diagnoses.

**Table 2:** Characteristics of 16 participating families

Characteristic	No. (%)
Patient Gender Male	9 (56%)
Family Country of Origin	
Mexico	4 (25%)
Guatemala	5 (31%)
Puerto Rico	1 (6%)
No answer	6 (38%)
Chief Complaint	
Fever and/or difficulty breathing	7 (44%)
Fever and/or cough	6 (38%)
Nasal congestion	1 (6%)
Fever	2 (12%)
Triage Acuity	
2 *(Most severe)*	3 (19%)
3	7 (44%)
4 *(Least severe)*	6 (38%)
Final ED Diagnosis	
Bronchiolitis	9 (56%)
Viral illness/Cold/Fever	4 (25%)
Croup	1 (6%)
Pneumonia	1 (6%)
Bilateral Otitis Media	1 (6%)
Method of Interpreter Used	
*In-Person*	6 (38%)
*Video*	5 (31%)
*Phone*	5 (31%)
Admitted	6 (37%)

### Thematic analysis

Four major themes emerged from the thematic analysis: (1) Uncertainty - fear and uncertainty regarding how to care for a child with distressing symptoms, (2) Communication – the presence of in-person interpreters enhanced communication, allowing parents to feel more informed, (3) System Burden- unfamiliarity with the U.S. healthcare system and lack of resources are additional sources of hardship and (4) Emotional Support - despite limited understanding, “successful” visits garnered confidence and provided parents with needed reassurance. Table 3 lists illustrative quotations representative of major themes and subthemes.

**Table 3:** Themes and subthemes with illustrative quotations

Themes and Subthemes, With Illustrative Quotations	
Theme or Subtheme	Illustrative Quotation
*Uncertainty*	
Complexity	“But that was just the fever but the thing I was worried the most about was his chest”
Concern	“Oh, I was worried about my little girl dying of the disease, so I wanted to get her to the emergency room quickly”
Confidence in one’s ability	“When he was home I couldn’t give him anything because if I gave him something I thought I could make things worse. I couldn’t give him just anything.”
*Communication*	
Communication during visit (intake, history)	“They examined him and they told me that he was very sick so they gave him oxygen and they told me that the doctor would come to examine him and tell me if he was going to be hospitalized or not. Half an hour later the doctor came and he told us that he needed to stay”
Informed	“Yes, they explained how everything was going to be done and I was ok with that”
Method of Communication	“Yes, having the in-person interpreter over there was better” “I felt comfortable because they had a face-to-face interpreter”
Understanding of diagnosis/Treatment plan	“I don’t speak English, I speak Spanish. I did understand everything. They had a, what’s it called? A translator, an interpreter. Something like that. “
*System Burden*	
Community resources	“Yeah, he has a social worker”
Familiarity with healthcare system	“So, it’s very different. I have been living here for like 2 years but the way they treat you here is very different compared to the way they treat you over there when you come to the emergency room with a child. In Puerto Rico, they would run a CBC test, and they would run lab tests, and ultrasound. And that’s not how it is here”
Navigating the System	“I haven’t found a way to get into health insurance”
Access	“Yes, she’s going to a clinic, well I take her to a clinic called Price Hill. It’s about 5 minutes from here” “We have to ask a guy to give a us a ride every day. We just call and they come take us”
Family member	“My sister-in-law told the girl to find a girl who spoke English so I could explain things to her”
*Emotional Support*	
Reassurance	“Thanks to God, everything was going good” “I’m just grateful for everything they did”
Physician’s authority	“I did expect something else, but I was fine with it, they’re the doctors, of course”
Trust	“They did everything my baby needed” “I felt confident in my doctors and they took away that doubt and that anguish”

1. Uncertainty: Fear and concern regarding how to care for a child with distressing symptoms:
  “I was worried about my little girl dying of this disease.” “Maybe if it was another case, my baby might have died, because we don’t know how to take care of the baby in the house.”

Participants reported that their children were experiencing various symptoms that were disconcerting. Parents were frightened by the symptoms and were unsure of their ability to care for their child at home. Some participants reported that they did not provide any treatment at home because they were inexperienced as a parent and thus not sure of the appropriate course of action. Others believed that if they provided interventions, they might make the situation worse. Furthermore, parents were fearful that if they did seek medical treatment, their limited English proficiency would affect their ability to effectively communicate with medical professionals. Nevertheless, their child’s distressing symptoms prompted parents to seek help anyway.

2. Communication: The presence of in-person interpreters enhanced communication, allowing parents to feel more informed:

“They explained how everything was going to be done and I was okay with that.” “Yes, having the in-person interpreter over there was better” “It did help me a lot because that way I was able to communicate through this person. I was able to explain everything. I was able to explain and say everything – everything that was going on with my son. And everything the doctor and the nurse wanted to tell me I was able to understand all of it through this person”

Participants reported that providers attempted to explain their child’s diagnosis and treatment plan; however, limited understanding of medical terminology was a barrier. Despite this issue, in-person interpreters lessened parents’ confusion and improved communication by translating parental concerns and describing procedures and tests discussed by the provider. The frequency of reported understanding was higher with in-person and video interpreters. To facilitate understanding, one participant reported that their provider showed a video depicting symptoms that would require medical attention. These measures (i.e., videos, pictures, infographics) made most parents feel informed of the plan of care and few reported feeling ignored and uninformed.

3. System Burden: Unfamiliarity with the U.S. healthcare system and lack of resources are additional sources of hardship:

“The way you are treated here is very different.” “I mean, for them to do something it has to be a life or death situation.” “For me yes; I haven’t found a way to get into health insurance” “I live alone and I am alone with my baby and so technically it is all me”

Parental expectations for care in the U.S. were affected by care they had previously received in their home country. Some parents believed that in order to receive the standard of care they are accustomed to in the PED, their child must be severely ill. Participants also reported having trouble making appointments with primary care pediatricians and limited accessibility to clinics that provide interpreters. Some parents listed social workers and family members accustomed to the system as sources of advice and assistance in coordinating care and accessing treatment. However, many participants reported social isolation and few community resources.

4. Emotional Support: Despite limited understanding, “Successful” visits garnered confidence and provided parents with needed reassurance:

“I felt confident in my doctors and they took away that doubt and that anguish.” *“*They did everything my baby needed.” “I loved how they [the interpreter] explained the results of the x-ray.”

Although parents did not completely understand their provider, many were satisfied with the visit. Most participants believed their providers adequately addressed their concerns and provided adequate care to their children. However, a few parents felt the appointment was rushed and their concerns were not taken seriously due to the mild nature of their child’s illness. Overall, participants trusted their providers and were calmed by their provider’s assurance about their child’s prognosis.

## Discussion

This study explored Latinx families’ lived experiences regarding the treatment of their children when presenting to a PED with respiratory symptoms within a NDA. This is extremely important for PED providers because they are treating patients at one point in time, without much, if any, foreknowledge of what led the families to their visit. Obtaining the clinical information, such as the child’s symptoms and clinical severity, is a streamlined process. However, exploring how families experienced their visit in the ED can be more difficult to assess, but can still be important information for providers. In this study, the major themes that emerged for all 3 interviewers included the fear of progression of illness due to the families’ uncertainty about the child’s clinical status, the importance of using in-person interpreters, unfamiliarity with the healthcare system, and the shift in perspective from lack of confidence to confidence in their ability to care for their child after the healthcare provider’s diagnosis and management in the PED.

This study reinforced the importance of having in-person and/or video interpreters, which is a relatively simple but vital aspect of a family’s experience when presenting to a PED for any health concern while facing a language barrier. Families felt more informed and reassured when a video or a combination of an in-person and video interpreter were used during the PED visit. It is known that the use of professional interpreters is associated with overall improvement in care for patients with limited English proficiency, with in-person or video interpretation leading to higher patient satisfaction compared to telephone interpretation [30, 31]. Prior research examining the Latinx population has shown that a lack of staff members of the same ethnic or language group is associated with barriers to health care [18, 32]. Although several prior research studies show that families may still have a degree of mistrust regardless of having access to an interpreter [33, 34], families in this study reaffirmed that they remained informed and reassured when they were given the opportunity to interact with an interpreter in-person or through a video.

Navigating the healthcare system within a NDA was another major theme among participants. Disparities in pediatric care may be due to a lack of access to primary care for Spanish-speakers [7]. Families often reported trying to visit primary care clinics first, but they were unable to get a visit with a Spanish-speaking provider or they did not know what to do if it was after regular business hours. These experiences support the literature that suggests increased PED utilization for routine sick visits among Latinx children, resulting in fewer interventions in the PED due to the higher frequency of low acuity visits [12, 33]. In contrast, patients among families within this study who reported difficulty with navigating the healthcare system had lower triage levels (higher acuity). Navigating the healthcare system can be more difficult among Latinx families within a NDA, which could lead to sicker patients going to the PED. Lower acculturation among ethnic groups has been shown to be associated with higher numbers of PED visits [32]. The increased difficulty for Latinx families within an NDA can lead to even higher numbers of PED visits and may influence the level of care needed in the emergency setting.

Another key issue highlighted in this study was the importance of educating the family about their child’s diagnosis and management in the PED. Families were often very grateful for the treatment they received even if the intervention was minimal such as nasal suctioning or providing an antipyretic. Although the need for extensive testing and interventions was low in this participant cohort, parents were very thankful and reassured for the care they received in the PED. This finding is interesting in the light of prior quantitative findings showing that Latinx patients facing language barriers receive increased PED charges and increased testing compared to patients without language barriers [13, 14]. Furthermore, it was not clear if parents always understood their child’s diagnosis or return precautions because of language barriers or health literacy levels. One family expressed appreciation for a short video that was shared with them before discharge that demonstrated an infant in respiratory distress as this ensured they understood appropriate return precautions. Nonetheless, families were reassured and more confident at the end of the encounter, regardless of whether the patient needed numerous tests or interventions in the PED.

There have been a few studies exploring Latino caregivers’ experiences within the PED [32, 35, 36]. However, no studies of similar methodology have been done within a NDA. A study within a PED in Texas was done with similar methodology to our study, and the researchers focused on children who presented to the PED with asthma symptoms [35]. Similar to our study, the researchers found themes that included uncertainty, importance of interpreter availability, and access to care. However, in our study, families reported the increased reliance of family members in their access to care. We also found that families valued the emotional support provided by the providers and staff, which may be important in a NDA in which there is typically not a large Latinx population.

There were several limitations that should be noted. Two of the three interviewers were not from the Latinx community, which could have impacted participants’ willingness to be open and honest. One interviewer did have to use an interpreter; however, the emergent themes did not differ among the 3 interviewers. Although data reached thematic saturation [23], the majority of the interviewed families were from the same country (e.g., Guatemala). Thus, findings should be interpreted with caution and may not apply to other Latinx populations. Lastly, although intentional in qualitative research, the study was conducted at a single site with non-probability sampling which limits the applicability of findings to other clinical settings.

## Conclusions

Our study identified four major themes among Latinx families within a PED of a NDA. All families were fearful of their child’s illness, but they appreciated being informed via an interpreter and felt reassured by the end of the PED visit. However, families did have difficulty navigating the healthcare system. Qualitative studies such as this study can help identify specific factors from the parents’ experiences that could be improved to reduce disparities related to language barriers. Further areas of intervention should focus on support beyond providing access to an interpreter, such as improving information delivery, and enhancing education on community resources for families with low English proficiency in the PED.

## Data Availability

Data cannot be shared publicly because of potentially identifying patient information. Data are available from the Division of Emergency Medicine at Cincinnati Children’s Hospital Medical Center (contact Dr. Mahabee-Gittens via melinda.mahabee-gittens@cchmc.org) for researchers who meet the criteria for access to confidential data.

## Abbreviations

PED: Pediatric Emergency Department
ED: Emergency Department
NDA: Nontraditional Destination Area
CCHMC: Cincinnati Children’s Hospital Medical Center
EHR: Electronic Health Record
